# Travelling for Health

**Published:** 1889-11-23

**Authors:** J. Bowles Daly


### "Travelling for Health."

By Dr. J. Bowles Daly.

The November fogs will drive many from town, sending them to Brighton, Hastings, and other places of resort. Many of our people regularly winter in Italy and the South of France, finding this southern climate highly beneficial to a delicate constitution. A few remarks from an old traveller may not be out of place.

In our modern life there is a great hunger for change, utterly unknown to our ancestors, who, for the most part, lacked the opportunity. This desire is healthy; any objections to travelling based upon our grandfathers' indifference to it might be urged with equal force against the penny postage or the pocket-handkerchief. We could do without either, and many do. Country people run up to town for relaxation, and townspeople escape to the country for the same purpose. Let the barrister loose from the court and chambers, and give the clerk a month's respite from the office—where will you find them in a week? On the top of Mount Rosa, or among the Cumberland lakes.

I put up one summer at an out-of-the-way *châlet* among the mountains of Spain, and found there an Irish Dissenting minister, an inspector of schools, and a London police magistrate in wide-awakes, to say nothing of an archdeacon in high-lows and a flannel shirt, discussing the shortest climb to a neighbouring peak.

The charms of travelling consist in its total change from daily drudgery, and the complete release from stale scenes. Of all travelling, that on the Continent is the most refreshing. Of course, it has its drawbacks. The voyage across the Channel is perhaps the worst if a man is a bad sailor, and few can keep their own that brief journey. The tourist's ignorance of any language besides his own may be another. This, however, is the slightest, for I believe there is hardly a spot anywhere in the globe where, if you shout in English, somebody will not come. But suppose he does not? I really think the sensation of not being understood is rather entertaining than otherwise; it makes the change you are seeking more complete. You can persist in a number of indulgences on the grounds of prohibitions being unintelligible. This is a subtle form of delight which is a novelty in itself, provided that it is not carried too far. If you fail to satisfy a succession of discontented *garçons* you have the great advantage of not understanding them. Their satire, both direct and indirect, may be withering, but you don't know it. They can't insult you except by gestures and grimaces, which are often worth the money to see instead of being the reward and consequence of a just economy. Then, again, if you are angry with yourself, what a relief to abuse the offender in the warmest language you can command—judiciously selecting the strongest adjectives. He is none the worse for it, while you are all the better. He does not understand a word, and you would have been sorry half an hour afterwards if he could. Since it takes two to make a quarrel and the rejoinder is more irritating than the original insult, all dangerous provocation is neutralised, and yet the excess of steam is blown off by both. Depend upon it, it is a great thing for a reply to be unintelligible. I offer a few pieces of advice worth more gold than the editor will give me for this; not many will take it, but it is true nevertheless. Don't harass yourself with plans; study maps and guides before you start; and get all the information you can from your friends. Once abroad, however, don't tie yourself to any routine. It is simply delicious to defy the arrangement of a campaign and revel in the consciousness of errant independence. People who draw out their route beforehand and try to follow it create a fund of reproachful memories.

Again, if you are not fond of churches or picture-galleries, don't bother about them. How often I pitied haggard groups who gaped listlessly along the galleries of the Pitti Palace and the Louvre. Why will a man who has no knowledge of such matters not have the courage to refuse to look at pictures or buildings? If a man does not delight in Westminster Abbey or the National Gallery, he is not likely to derive much gratification from what he can find abroad. There is an endless fund of gratification that requires no culture, if he will draw upon it, in merely prowling about with his eyes open. Let him only feel in his conscience that he is not playing the hypocrite, and he will find everything that is novel more or less interesting.

Avoid big hotels and learn to smile at bills. You know what an English or Scotch innkeeper is? Well, his neighbour on the Continent is a brother. If you suffer little extortions to annoy you, you had better stay at home and stick to the shop. Don't travel first-class abroad, or you will find yourself in the company of English invalids, and, if possible, don't try to make yourself out to be a bigger man than you are at home. A morose silence stamps the Englishman.

I can't conclude without a hint about food. In dining at *tables d'hôte* always take something of what is offered. Otherwise, when you have missed several opportunities, you may all at once find yourself left with nothing between you and starvation but a tureen of stewed plums followed by a toothpick. *Table d'hôte* dinners have a trick of sudden finality; and remember there is not even bread and cheese wherewith to fill the vacuum which you abhor. The secret of all travelling is to be unfettered—to cast off restraints which are necessary at home—and move about when and where you like in your own foolish easy way. This enables body and mind to uncoil themselves without labour or strain, and then you get what you want—namely, recreation.

